# USP22 Promotes Osteosarcoma Progression by Stabilising β‐Catenin and Upregulating HK2 and Glycolysis

**DOI:** 10.1111/jcmm.70239

**Published:** 2024-12-11

**Authors:** Shenliang Chen, Xin Hu, Xuan Yi, Xueqiang Deng, Ting Xiong, Yanghuan Ou, Shuaigang Liu, Chen Li, Xiaohua Yan, Liang Hao

**Affiliations:** ^1^ Department of Orthopedics, the 2nd Affiliated Hospital, Jiangxi Medical College Nanchang University Nanchang China; ^2^ University of Nanchang Nanchang China; ^3^ Jiangxi Pingxiang People's Hospital Pingxiang China; ^4^ Department of Biochemistry and Molecular Biology, School of Basic Medical Sciences Nanchang University Jiangxi Medical College Nanchang China

**Keywords:** β‐catenin, glycolysis, growth, hexokinase 2, osteosarcoma, USP22

## Abstract

Osteosarcoma is a primary malignancy that is difficult to treat and is prone to developing resistance to chemotherapy. As such, it is necessary to continuously explore novel therapeutic targets. Ubiquitin‐specific protease 22 (USP22) is an ubiquitin‐specific protease that has been demonstrated to have potent carcinogenic effects on a variety of cancers and is involved in several biological processes. Studies have demonstrated that reprogramming of glucose metabolism is a major factor in the development and progression of osteosarcoma, and that USP22 is strongly associated with the metabolism of glucose in osteosarcoma. However, it is still unknown how precisely USP22 works in osteosarcoma. To further elucidate the expression and specific molecular mechanisms of USP22 in osteosarcoma. The results of Western blot analysis and quantitative reverse transcription polymerase chain reaction (qRT‐PCR) showed that the expression of USP22 in osteosarcoma tissues was significantly higher than that in adjacent healthy tissues. In addition, the expression of USP22 promotes the proliferation of osteosarcoma cells in a glycolytic dependent manner both in vitro and in vivo, while the knockout of USP22 is the opposite. In addition, USP22 knockout reduced the protein expression of β‐catenin and hexokinase 2 (HK2) in osteosarcoma cells. In addition, the regulation of HK2 expression induced by USP22 depends on β‐catenin. Mechanistically, USP22 regulates HK2 by deubiquitination and stabilising the expression of β‐catenin, thereby controlling glycolysis in osteosarcoma cells.

## 1 Introduction

Osteosarcoma is a primary bone tumour characterised by a poor prognosis, high aggressiveness, early metastasis and a high incidence in children and adolescents [1, 2]. Although recent advances in adjuvant and neoadjuvant chemotherapy have improved the quality of life and survival rate of patients with osteosarcoma. However, patients will inevitably experience adverse effects from chemotherapy medicines, and the altered bone microenvironment encourages early metastasis and a high rate of recurrence in osteosarcoma as well as the development of self‐resistance mechanisms [3, 4]. Frequently, the outcomes of the treatment are inadequate [5, 6]. Therefore, early diagnosis and efficient treatment are crucial to improve patients' outcome and survival rate. Accordingly, identifying the precise molecular pathways underlying the aetiology of osteosarcoma and developing more potent therapeutic approaches are crucial.

Aerobic glycolysis plays a crucial role in various tumours [7, 8] and is associated with the rapid proliferation and advancement of tumour cells, as demonstrated by the ‘Warburg hypothesis’ (also known as the ‘Warburg effect’) [9, 10]. Therefore, glycolysis is recognised as a marker of tumour cells. Research findings indicate that HOXA1 controls the transcriptional activity of PGK1 and ENO1, augments glycolysis in cervical cancer cells and facilitates the disease's advancement [11]. Although the ATP production efficiency of the glycolytic pathway is low, its ATP production rate is nearly 100 times that of mitochondrial oxidative phosphorylation, which drives glucose metabolism in tumour cells [12, 13]. For example, research by Bartman et al. [14] shows that in human xenografts derived from pancreatic cancer, lung cancer and colorectal cancer, there is a high level of glycolysis, while oxidative phosphorylation is significantly inhibited. In addition, studies have shown that fascin stimulates YAP1's binding to the TEAD1/4 binding motif, which is situated upstream of PFKFB3's transcription start site. This binding activates PFKFB3's transcription, increases PFKFB3's expression and glycolysis, and encourages the growth and spread of lung cancer [15]. Also, in pancreatic cancer, FOXD1 directly stimulates SLC2A1 transcription, suppresses SLC2A1 degradation via an RNA‐induced silencing complex, upregulates GLUT1 expression and ultimately facilitates the growth, invasion and metastasis of pancreatic cancer cells by controlling aerobic [16]. Additionally, glycolysis increases the resistance of tumour cells to ischemia and hypoxia. Based on these studies, it can be concluded that tumour cells prefer glycolysis to oxidative phosphorylation [17]. Therefore, studying the specific molecular mechanisms underlying glycolysis in osteosarcoma is a promising research direction.

Three enzymes—ubiquitin ligase (E3), ubiquitin activating enzyme (E1) and ubiquitin binding enzyme (E2)—are involved the ubiquitination reaction. A crucial factor in substrate selection is E3 [18], while deubiquitinase (DUB) is involved in protein deubiquitination, both of which jointly regulate protein degradation and stability [19, 20]. Studies have verified a strong correlation between DUB and cancer incidence [21, 22]. DUB has recently emerged as a possible target for numerous tumour therapies [23]. Ubiquitin‐specific protease 22 (USP22), a member of the DUB family, plays various roles in the physiological functions of tumour cells, including DNA damage repair, cell proliferation, apoptosis and tumour stem cell maintenance and metabolism [24, 25, 26]. USP22 is highly expressed at the mRNA and protein levels in 14 common solid tumours, including pancreatic cancer, gastrointestinal tumours and liver cancer [27]. In breast cancer cells, USP22 deubiquitinates and stabilises c‐Myc to promote breast cancer progression [28]. Additionally, USP22 stabilises the expression of cyclin D1 and B1 via deubiquitination, thereby promoting the development and progression of colorectal cancer [29, 30]. Furthermore, current research indicates that USP22 facilitates the glycolysis of osteosarcoma [31]. The glycolysis precise molecular process underlying USP22 involvement in osteosarcoma remains unclear despite the fact that the protein's significance in osteosarcoma has been partially revealed more research is necessary to determine this.

HK has a high affinity for glucose and catalytic activity at both the N‐ and C‐termini, regulating glucose metabolism [32]. HK is an isoenzyme classified into five subtypes: HK1, HK2, HK3, HK4 and HKDC1, among which HK2 is the most active isoenzyme and is mainly found in musculoskeletal and cardiac cells. Hexokinase (HK) is an essential rate‐limiting enzyme that catalyses glucose metabolism [33]. Genetic anomalies in HK significantly affect the development and progression of several clinical diseases. Notably, HK2 is highly expressed in colorectal, liver and breast cancers [34, 35, 36]. Moreover, HK2 knockdown inhibits glycolysis and the proliferation of liver cancer cells [35]. HK2 regulates cell autophagy and death by performing functions that are unrelated to its enzymatic activity. However, HK2 activation in most tumour cells promotes metabolism and aerobic glycolysis [37]. Additionally, prior research has demonstrated that the interaction between RBM15 and circ‐CTNNB1 in osteosarcoma stimulates the growth of the tumour by promoting the expression of HK2 [7]. Nevertheless, there is still a deficiency in the HK2 regulation system in osteosarcoma. Therefore, further investigation is necessary to elucidate the precise molecular mechanism by which HK2 controls glycolysis in osteosarcoma.

A key molecule in the Wnt/β‐catenin pathway is β‐catenin. Activation of the Wnt/β‐catenin pathway increases the nuclear accumulation of β‐catenin, which attaches to TCF/LEF family members, initiating downstream target gene transcription [38]. Research evidence indicates that the activation of the Wnt/β‐catenin pathway is involved in tumour and nontumor disorders, including tumour metabolism, immunity, growth and metastasis [39]. When β‐catenin is inactivated, it becomes more phosphorylated and is broken down by the ubiquitin proteasome pathway [40]. However, little is known about the DUB involved in β‐catenin regulation. USP22, an ubiquitin‐specific protease, contributes to the degradation of targeted proteasomes and the polyubiquitination of proteins. Presently, it is uncertain whether USP22 plays a role in β‐catenin ubiquitination in osteosarcoma.

Therefore, this study aimed to investigate the expression, role and potential mechanism of USP22 in osteosarcoma. Specifically, we examined the role of USP22 expression in glycolysis and the proliferation of osteosarcoma cells using molecular techniques.

## 2 Materials and Methods

### 2.1 Organisational Samples

All normal bone and osteosarcoma tissue samples were obtained from Nanchang University's First and Second Affiliated Hospitals. Informed consents were obtained from the patients, and the study was approved by the Nanchang University Ethics Committee. A portion of the sample was stored in a refrigerator at −80°C for protein immunoblotting and quantitative reverse transcription polymerase chain reaction (qRT‐PCR), while the remaining portion was fixed in 4% paraformaldehyde for immunohistochemical analysis.

### 2.2 Cell Lines and Culture

Human osteosarcoma cell lines (including MG‐63, 143 B, U2‐OS and HOS) and normal human osteoblast (hfoBI‐19; control) were obtained from the American Type Culture Collection (ATCC). 143B and HOS cells were cultured in improved Dulbecco's modified Eagle medium (DMEM); MG‐63 cells were cultured in MEM; and hfoBI‐19 cells were cultured in DMEM/F12. All media were prepared in complete culture medium containing 10% FBS (fetal bovine serum), 1% streptomycin and penicillin. All cells were cultured at 37°C under a 5% CO_2_ atmosphere in a cell incubator.

### 2.3 Real Time Quantitative PCR

Total RNA was extracted from osteosarcoma tissues and cells using TRIzol reagent (TaKaRa, Japan) and reverse‐transcribed to generate cDNA using a PrimeScript RT kit (TaKaRa, Japan), according to the manufacturer's instructions. PCR amplification was on a BIO‐RAD C1000 Thermal Cycler using the SYBR Premium ExTaq II kit (BIO‐RAD) and specific primers. Three iterations of the experiment were conducted, and the level of gene expression was calibrated to the GADPH level. The following primer pairs were utilised: USP22, 5′‐CACCGGACAGTCCAACAAATG‐3′ and 5′‐GCATCGTCAACTTGAACACA‐3′; β‐catenin, 5′‐AGCGGTTAGTCACTGG‐3′ and 5′‐AGTCATTGCATGCATACTGTCCAT‐3′; HK2, 5′‐TGAGGTCTGATGCGGTGG‐3′ and 5′‐TGGCCTTTTTTTTTCCTTGATGC‐3′; and GAPDH, 5′‐GGTGTGGACCATGAGATGAGTATGA‐3′ and 5′‐GAGTCCTTCACGATACAAG‐3′.

### 2.4 Western Blot Analysis

Briefly, osteosarcoma tissue and cells were extracted using RIPA lysis buffer, followed by the addition of PMSF to prevent protein lysis and centrifugation at 13,000 rpm for 10 min to obtain the supernatants. Equal amounts of protein were separated using 10% sodium dodecyl sulfate‐polyacrylamide gel electrophoresis and transferred to PVDF membranes (Millipore, America). Thereafter, the membranes were blocked using 5% skim milk for 2 h, followed by overnight incubation with primary antibodies against USP22 (1:1000, Abcam), β‐catenin (1:1000, Abcam) and HK2 (1:1000, Abcam) at 4°C and further incubation with appropriate anti mouse/rabbit secondary antibodies at 25°C for 2 h. After three washes with 1 × TBST, the immune response band was observed using a chemiluminescence reagent kit (Transgen Biotech, China).

### 2.5 shRNA Plastics and Overexpression Constructions

shRNA encoding USP22 or β‐catenin and overexpression plasmids encoding HK2 or USP22 were purchased from Genepharma in Shanghai, China. The following shRNA sequences were employed in this investigation: USP22 shRNA (5′‐GCAUCAUAGACCAGAUCUUTT‐3′), β‐catenin shRNA (5′‐ACATCGAAGACTCTACAAT‐3′). Osteosarcoma cells were transfected with overexpression and shRNA plasmids using Lipofectamine 3000 (Invitrogen), according to the manufacturer's instructions. Successful transfection was confirmed at both the mRNA and protein levels. Osteosarcoma cells stably expressing USP22 or shUSP22 were screened using purithromycin (1–5 μg/mL, Sigma).

### 2.6 Cell Growth Assay

EdU assay: Briefly, 2 × 10^4^ cells were seeded into a single well of a 96‐well plate containing 100 μL of complete culture medium. After 24 h, the cells were incubated with EdU reagent according to the manufacturer's instructions, and cell proliferation was measured using a fluorescence microscope (Thermo Fisher Scientific, America).

CCK8 assay: Briefly, 5 × 10^3^ cells were seeded into a single well of a 96‐well plate containing 100 μL of complete culture medium. After 24 h, culture medium was replaced with a full culture medium containing CCK8 reagent. The absorbance was measured at 450 nm after 0, 24, 48 and 72 h using an enzyme‐linked immunosorbent assay (ELISA). Three duplicate secondary wells were used for each experiment.

Colony formation experiment: Osteosarcoma cells stably expressing USP22 or shUSP22 were seeded into six‐well plates (1.5 × 10^3^ cells/well), and the medium was changed every 3 days. After 2 weeks, the cells were fixed with 4% paraformaldehyde, stained with 0.5% crystal violet solution and photographed.

### 2.7 Flow Cytometry Assay

To assess apoptosis, cells (1 × 10^6^) were stained with Annexin V fluorescein isothiocyanate and propidium iodide apoptosis detection kit (BD Biosciences) according to the manufacturer's instructions. After staining using BD FACS Melody flow cytometry, the percentage of early and late apoptotic cells was assessed.

### 2.8 Determination of Tumorigenicity

All animal experimentations were approved by the Animal Ethics Committee of Nanchang University and adhered to the National Institutes of Health's Animal Care Guidelines. Briefly, shNC‐143B and shUSP22‐143B cells (1 × 10^6^ each) were subcutaneously transplanted into female nude mice (6‐week‐old). Tumour volume was measured every 5 days starting from the date of transplantation. After 5 weeks, mice were dissected and the tumours were weighed and photographed.

### 2.9 Extracellular Acidification Rate and Oxygen Consumption Rate

Mitochondrial respiration and glycolytic rates were measured using the XF Cell Mito Stress Test Kit and Glycolysis Stress Test Kit (Seahorses Bioscience), respectively. Extracellular acidification rate (ECAR) was analysed using the extracellular flux analyser XF96 (Seahorses Bioscience, Billerica, MA, USA).

### 2.10 Co‐Immunoprecipitation and In Vitro Ubiquitination Assay

Briefly, cells were treated with Co‐immunoprecipitation (Co‐IP) cell lysis solution on ice for 30 min, followed by the addition of IP buffer (500 μL) and A/G agarose beads. Thereafter, the cells were incubated with 2 μL of the first antibody overnight at 4°C, followed by centrifugation to collect agarose beads. After washing, the beads were resuspended in 20 μL of IP buffer, followed by the addition of protein buffer and boiling for 10 min. The samples were analysed using 10% sodium dodecyl sulfate‐polyacrylamide gel electrophoresis. Osteosarcoma cells were transfected with shUSP22 or USP22 plasmids. After 48 h, the cells were treated with MG132 (15 μmol/L) for 5 h. Immunoblotting was performed on the cell lysates using antibodies against Ub and β‐catenin.

### 2.11 Statistical Analyses

All statistical analyses were performed using the software GraphPad Prism 8.0 (GraphPad, San Diego, USA). Data are expressed as mean ± SD. Significant differences between two groups were determined using the Student's *t*‐test, while comparisons between more than two groups were performed using single‐factor analysis of variance (ANOVA). Statistical significance was set at *p* < 0.05.

## 3 Results

### 3.1 USP22 Expression Is Upregulated in Osteosarcoma Cells and Tissues

**FIGURE 1:**
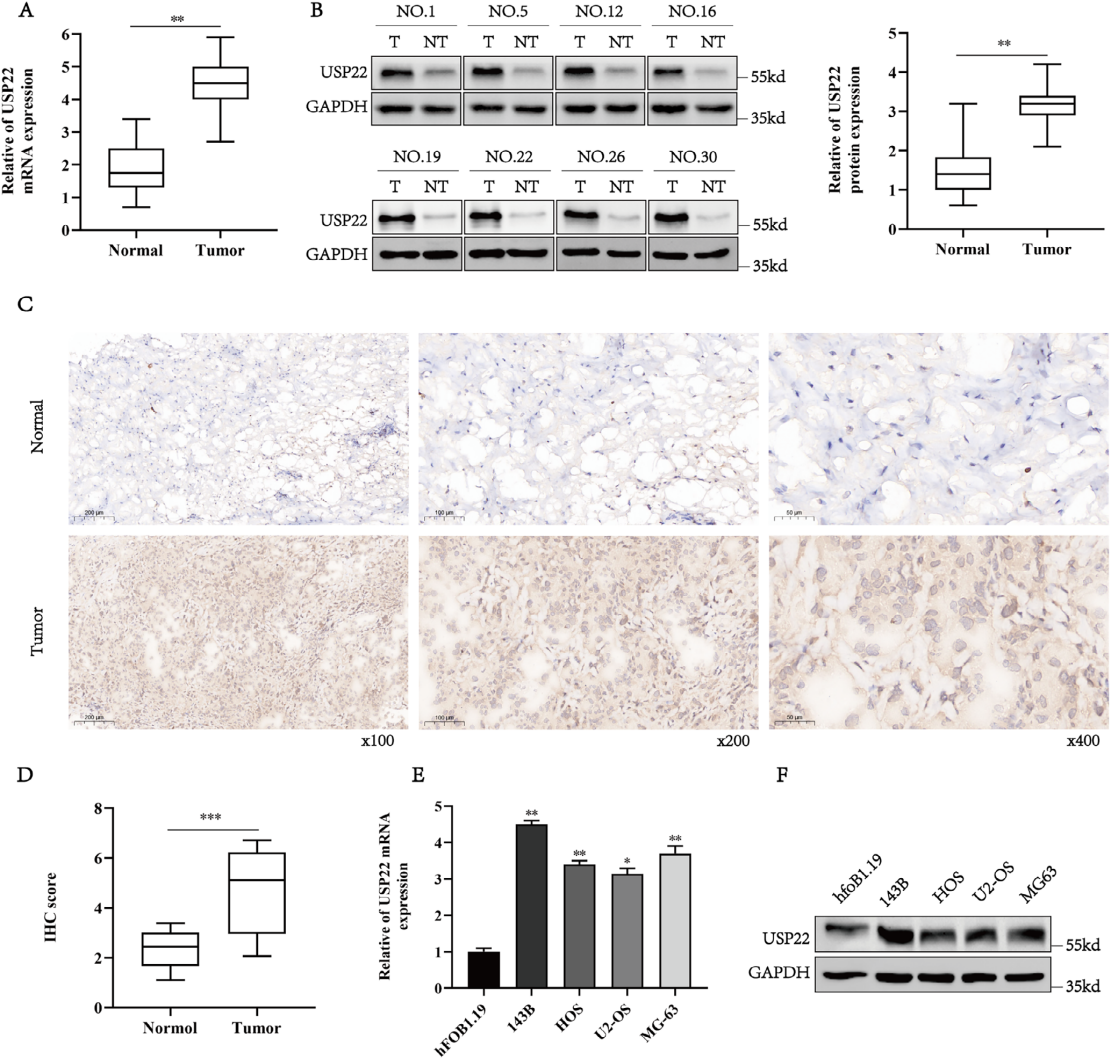
Relative USP22 expression in osteosarcoma cells and tissues. (A) Relative mRNA levels of USP22 in 30 pairs of osteosarcoma (OS) and corresponding non‐tumour tissues, assessed by qRT‐PCR analysis. *p < 0.05. (B) Determination and quantification of USP22 protein levels in osteosarcoma (OS) and corresponding non‐tumour tissues by western blotting assay. GAPDH was used as a loading control. (C, D) Representative images (C) and quantification (D) of USP22 staining in 30 paired osteosarcoma (OS) and noncancer tissues. *p < 0.05. (E, F) mRNA and protein levels of USP22 in osteosarcoma (OS) cells (143B, HOS, MG‐63, U2‐OS) and the immortalised normal cells (hfoBI‐19) line.

To investigate the role of USP22 in osteosarcoma, we compared the expression levels of USP22 in osteosarcoma tissue and neighbouring tissues and found that USP22 mRNA expression was significantly higher in osteosarcoma tissue than in neighbouring tissues (Figure 1A). Additionally, protein blotting showed that USP22 expression was significantly upregulated in matching tumour tissues than in neighbouring tissues (Figure 1B). Moreover, immunohistochemical (IHC) indicated that osteosarcoma tissues had considerably elevated USP22 levels (Figure 1C,D). Furthermore, qRT‐PCR and western blot analyses confirmed that USP22 expression was significantly higher in osteosarcoma cell lines (143B, HOS, MG‐63 and U2‐OS) than in the normal bone cell line hfoBI‐19 (Figure 1E,F).

### 3.2 USP22 Stimulates the Proliferation of Osteosarcoma Cells In Vitro and In Vivo

**FIGURE 2:**
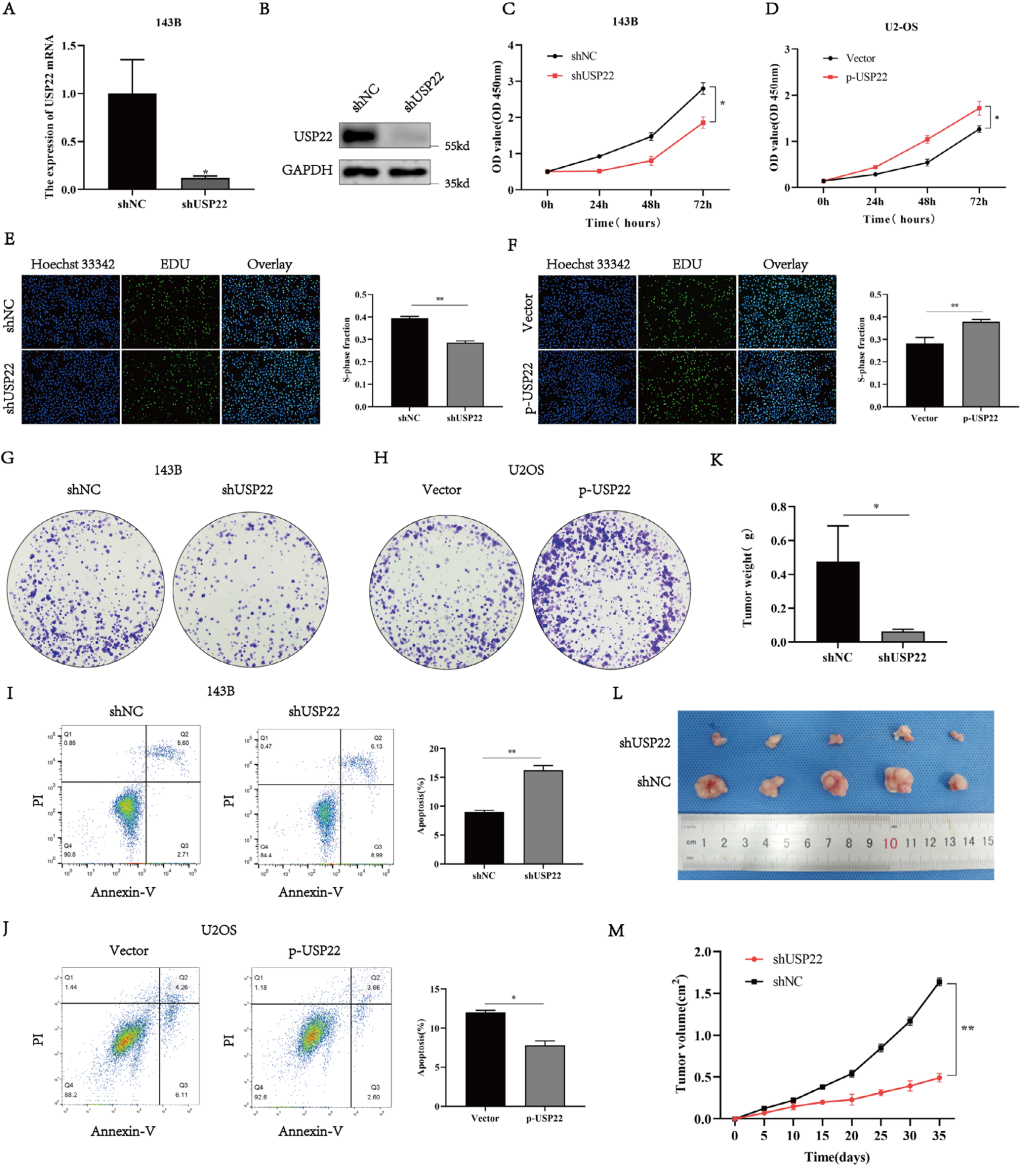
Effects of USP22 on osteosarcoma growth in vitro and in vivo. (A, B) the mRNA (A) and protein (B) levels of USP22 in 143B cells after transfection with shUSP22 or shNC (control). (C, D) CCK‐8 assay showing proliferation of OS cancer cells following overexpression (right) or knockdown (left) of USP22. *p < 0.05. (E, F) Representative images (left) and quantification (right) of EDU assays of osteosarcoma cells transfected with p‐USP22 or shUSP22. Scale bar, 50 μm. *p < 0.05. (G, H) Representative images of colony formation assays of osteosarcoma (OS) cells transfected with shUSP22 (G) or p‐USP22 (H). (I, J) The apoptosis rate of OS cells was detected by flow cytometry and was significantly increased in the shUSP22 cells but decreased in the p‐USP22 cells. (K–M) 143B/shUSP22 cells were subcutaneously injected into nude mice, and tumour volumes were measured on the indicated days; at the experimental endpoint, tumours were dissected, photographed and weighed (*n* = 5, *p < 0.05).

To elucidate the role of USP22 on osteosarcoma cell proliferation, we generated 143‐B cells stably expressing USP22 shRNA (Figure 2A,B) and U2OS cells overexpressing USP22 (Figure S1A,B). CCK‐8, EdU and colony formation assays showed that USP22 knockdown significantly decreased the proliferation ability of 143B cells, whereas USP22 overexpression significantly increased the proliferation of U2OS cells (Figure 2C–H). Furthermore, we examined the effect of USP22 on osteosarcoma cell apoptosis using flow cytometry and found that USP22 knockdown significantly increased 143B cell apoptosis compared with that in the shNC treatment group (Figure 2I). In contrast, USP22 overexpression significantly decreased U2OS cell apoptosis compared with that in the control group (Figure 2J).

To investigate the effect of USP22 on tumour growth in vivo, shNC‐143B and shUSP22‐143B cells were subcutaneously transplanted into female nude mice. From the day of injection until the conclusion of the fifth week, measure the tumour's size every 5 days. Compared with that in the control group (shNC‐143B), tumour growth and weight were significantly lower in mice injected with shUSP22‐143B cells after 5 weeks (Figure 2K–M). Overall, these results indicate that USP22 promotes the proliferation of osteosarcoma cells both in vivo and in vitro.

### 3.3 USP22 Activates Aerobic Glycolysis in Osteosarcoma Cells

**FIGURE 3:**
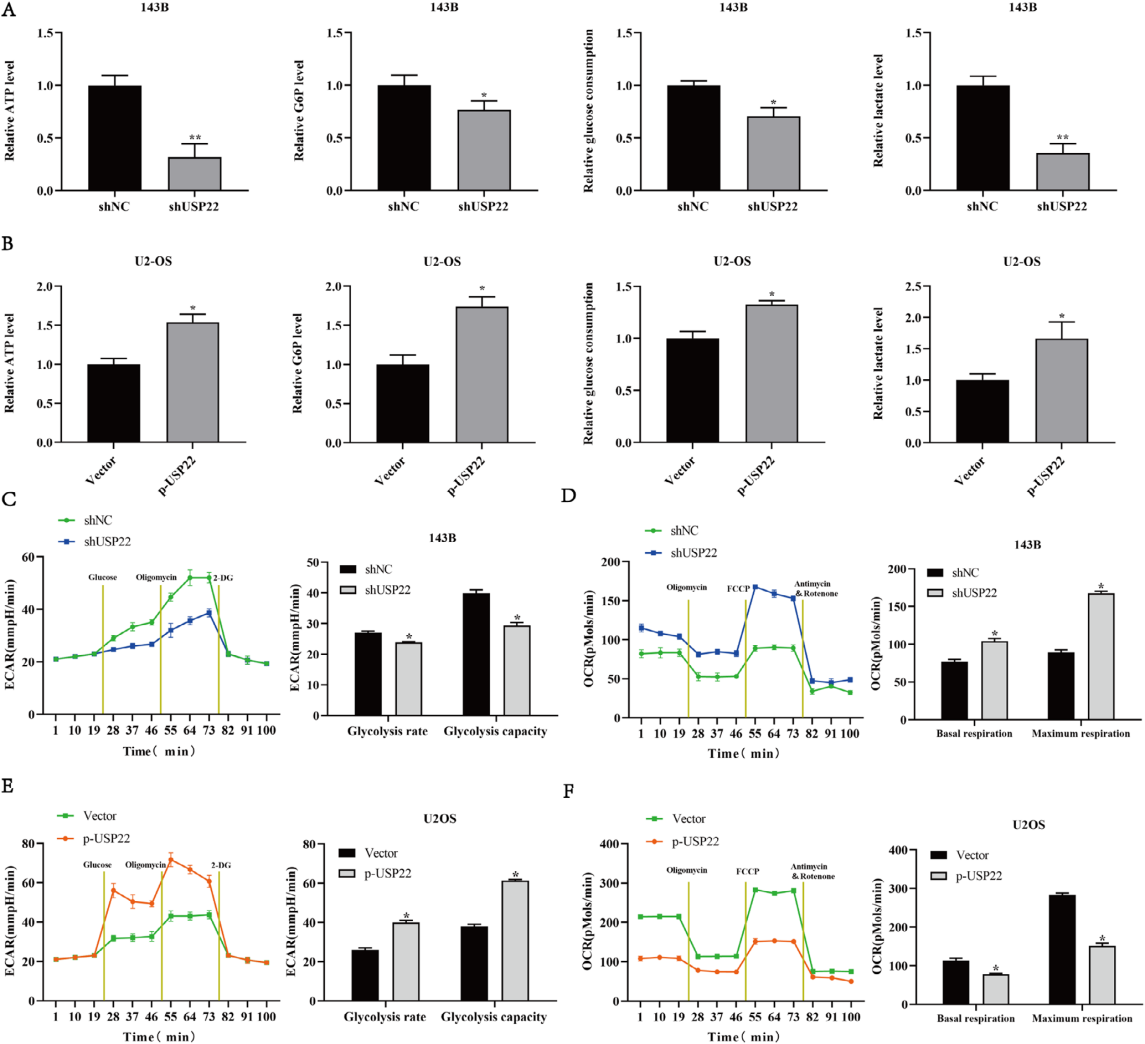
USP22 promotes aerobic glycolysis in osteosarcoma cells. (A, B) ATP levels, Cellular G6P levels, glucose consumption and lactate production in 143B/shUSP22 cells (A) or U2OS/p‐USP22 cells (B). Three independent experiments were performed. *p < 0.05 versus control. (C, E) ECAR data showing the glycolytic rate and capacity in USP22‐silenced (C) or USP22‐overexpressing (E) osteosarcoma cells. Glucose (10 mM), the oxidative phosphorylation inhibitor oligomycin (1.0 μM) and the glycolytic inhibitor 2‐deoxyglucose (2‐DG, 50 mM) were sequentially injected into each well at the indicated time points. All measurements were normalised to the cell number calculated using crystal violet assay at the end of the experiment. *p < 0.05 versus control. (D, F) OCR results showing the basal respiration and maximum respiration in 143B/shUSP22 cells (D) or U2OS/p‐USP22 cells (F). Oligomycin (1.0 μM), the mitochondrial uncoupler carbonyl cyanide p‐trifluoromethoxy phenylhydrazone (FCCP, 1.0 μM) and the mitochondrial complex I inhibitor rotenone plus the mitochondrial complex III inhibitor antimycin A (Rote/AA, 0.5 μM) were sequentially injected. All measurements were normalised to the cell number calculated using crystal violet assay at the end of the experiment. *p < 0.05 versus control.

Aerobic glycolysis is widely recognised as a characteristic metabolic pathway in osteosarcoma. Therefore, we investigated the effects of USP22 on glucose metabolism in osteosarcoma cells. USP22 knockdown decreased ATP and glucose‐6‐phosphate (G6P) levels, glucose intake and lactate generation in 143B cells (Figure 3A), whereas the opposite effect was observed in U2OS cells overexpressing USP22 (Figure 3B). Additionally, we assessed the ECAR in osteosarcoma cells to determine the impact of USP22 on glycolysis. USP22 knockdown significantly decreased glycolysis rate in 143B cells (Figure 3C), whereas the opposite effect was observed in U2OS cells overexpressing USP22 (Figure 3E). Moreover, USP22 overexpression significantly decreased oxygen consumption rate (OCR) in U2OS cells, whereas USP22 knockdown increased OCR in 143B cells (Figure 3D,F). Collectively, these results suggest that USP22 promotes aerobic glycolysis in osteosarcoma cells.

### 3.4 USP22 Positively Regulates HK2 Protein Levels

**FIGURE 4:**
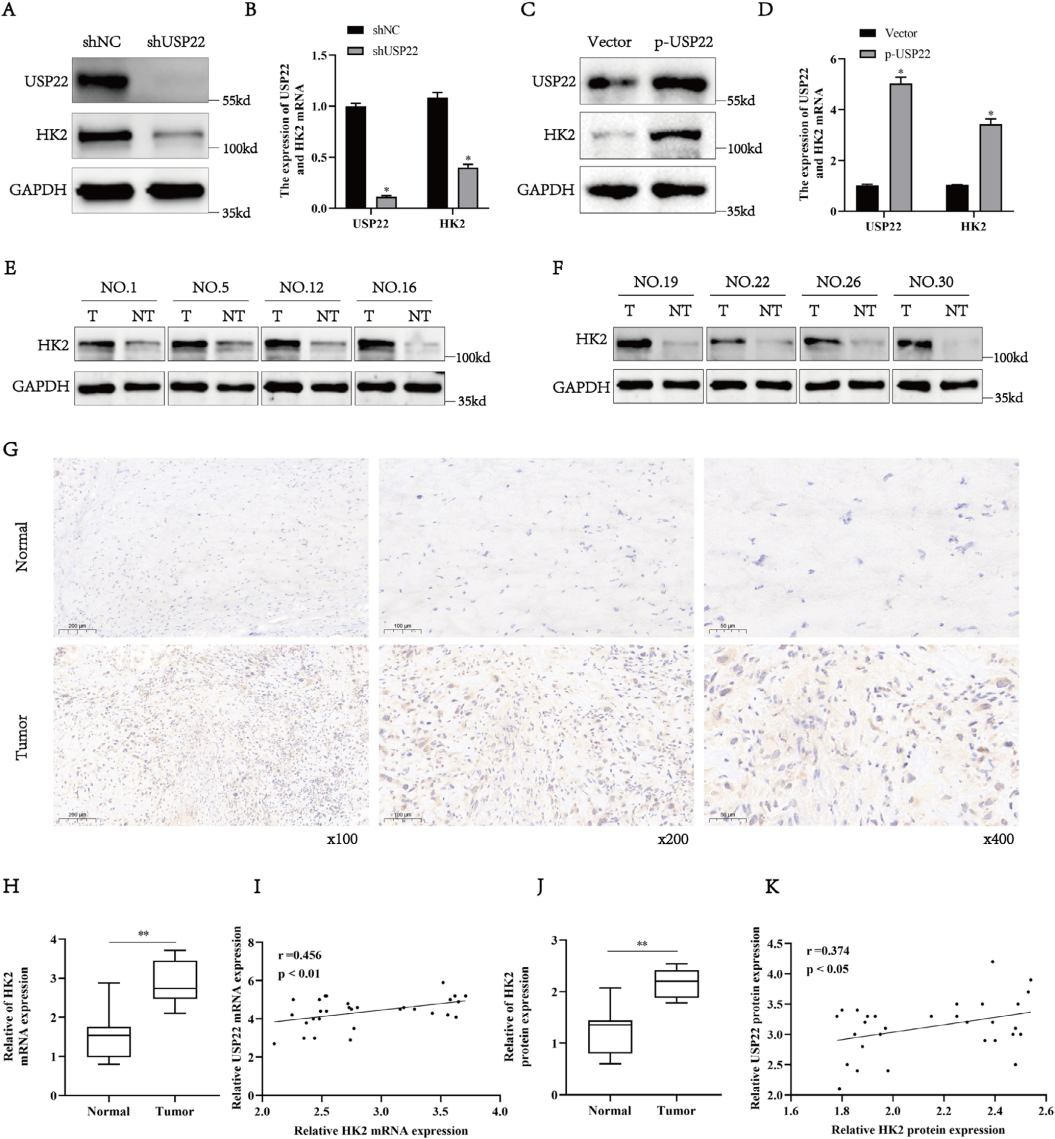
Stable knockdown of USP22 decreased HK2 expression in osteosarcoma cells. (A, B) Western blotting and qRT‐PCR analyses of HK2 expression levels in 143B cells stably transfected with shNC or shUSP22 plasmid. *p < 0.05. (C, D) Western blotting and qRT‐PCR analyses of HK2 expression levels in U2OS cells stably transfected with control vector or p‐USP22 plasmid. *p < 0.05. (E, F) Determination of HK2 protein levels in osteosarcoma tissues (*n* = 30) and paired non‐tumour tissues (*n* = 30) by western blotting. GAPDH was used as a loading control. (G) Representative images of HK2 staining in 30 paired osteosarcoma (OS) and noncancer tissues. (H) Determination of HK2 mRNA levels in osteosarcoma tissues (*n* = 30) and paired non‐tumour tissues (*n* = 30) by qRT‐PCR. (I) Scatter plots of USP22 and HK2 mRNA expression in osteosarcoma. (J) Quantification of HK2 protein levels in osteosarcoma (OS) and corresponding non‐tumour tissues by western blotting assay. (K) Scatter plots of USP22 and HK2 protein expression in osteosarcoma.

Research has demonstrated that HK2 is involved in aberrant metabolism in tumour cells and serves as a key regulatory factor in tumour development. Therefore, we speculated that USP22 can regulate HK2 expression. To confirm our hypothesis, we examined HK2 protein and mRNA expression in osteosarcoma cells following USP22 knockdown and overexpression. USP22 knockdown significantly decreased HK2 protein and mRNA expression in 143B cells (Figure 4A,B). In contrast, USP22 overexpression in U2OS cells increased HK2 protein and mRNA expression (Figure 4C,D). Additionally, we examined HK2 protein and mRNA expression in 30 osteosarcoma tissue samples. Compared with that in adjacent tissues, HK2 protein (Figure 4E,F,J) and mRNA (Figure 4H) expression was significantly higher in osteosarcoma tissue, which was confirmed by immunohistochemical staining (Figure 4G). Moreover, scatter plots showed a strong positive correlation between USP22 and HK2 mRNA expression levels (Figure 4I). Similarly, USP22 and HK2 protein expression levels showed a strong positive association (Figure 4K). Overall, these results indicate that USP22 positively regulates HK2 expression.

### 3.5 HK2 Mediates USP22‐Induced Aerobic Glycolysis in Osteosarcoma Cells

**FIGURE 5:**
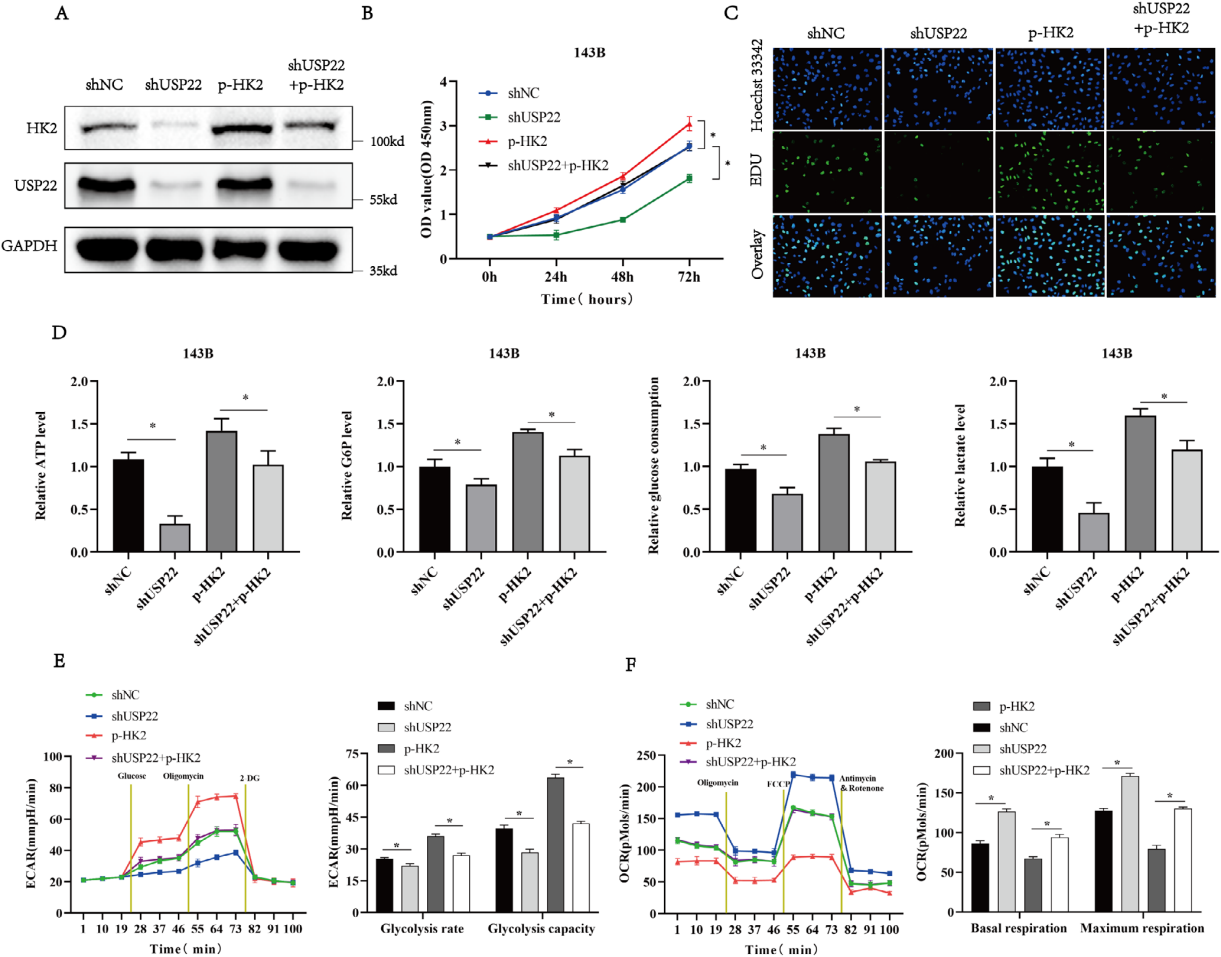
Tumour‐suppressive effects of USP22 silencing in osteosarcoma cells partially reversed by HK2 overexpression. (A) Western blotting of USP22 or HK2 in 143B cells stably transfected with shUSP22 in the presence or absence of p‐HK2. (B) CCK‐8 assays showing proliferation capacity of 143B cells stably transfected with shUSP22 in the presence or absence of p‐HK2. *p < 0.05. (C) Representative images of EdU assays of 143B cells stably transfected with shUSP22 in the presence or absence of p‐HK2. (D) ATP levels, Cellular G6P levels, glucose consumption and lactate production in 143B cells stably transfected with shUSP22 in the presence or absence of p‐HK2. *p < 0.05. (E) ECAR of USP22‐silenced 143B cells with and without HK2 overexpression. *p < 0.05. (F) OCR values of USP22 silenced 143B cells with and without HK2 overexpression. *p < 0.05.

Given that USP22 is positively associated with HK2, we speculate that USP22 modulates glycolysis in osteosarcoma cells by regulating the expression of HK2. To confirm this hypothesis, we overexpressed HK2 in 143B cells transfected with shUSP22 (Figure 5A). Additionally, we investigated the effect of HK2 overexpression on the proliferation of shUSP22‐transfected 143B cells using CCK‐8 and EdU assays. USP22 knockdown‐induced decrease in 143B cell proliferation was countered by HK2 overexpression (Figure 5B,C, Figure S2A). Similarly, HK2 overexpression partially reversed the USP22 knockdown‐induced decrease in ATP, G6P, glucose consumption and lactate levels in 143B cells (Figure 5D). Additionally, HK2 overexpression partially reversed the USP22 knockdown‐induced decrease in ECAR in 143B cells (Figure 5E). Moreover, HK2 overexpression suppressed the USP22 knockdown‐induced increase in OCR in 143B cells (Figure 5F). Collectively, these results suggest that HK2 mediates glycolysis in osteosarcoma cells via USP22.

### 3.6 USP22 Regulates the Expression of HK2 by Activating β‐Catenin in Osteosarcoma Cells

**FIGURE 6:**
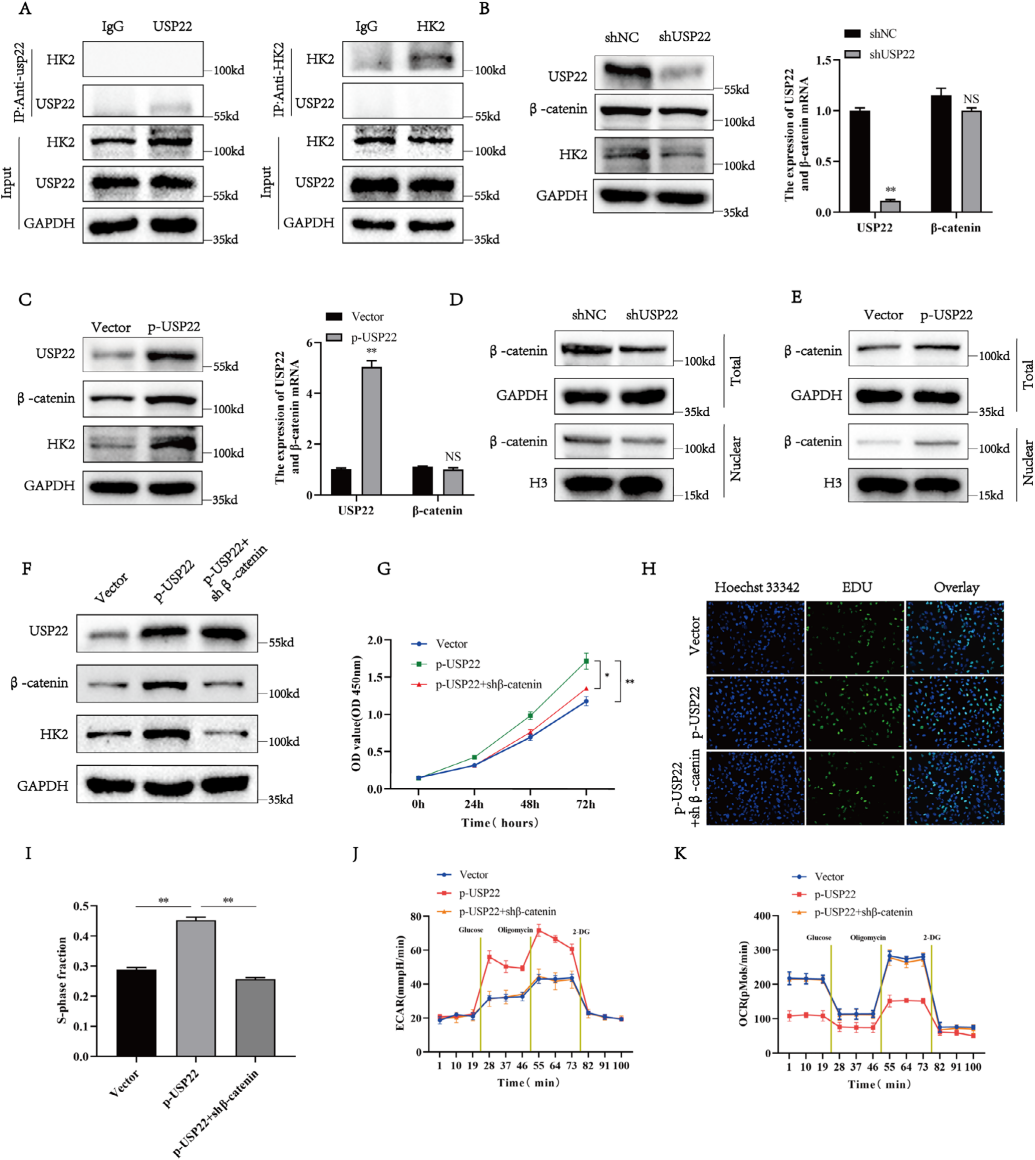
USP22 regulates HK2 expression through β‐catenin in osteosarcoma. (A) Co‐immunoprecipitation (Co‐IP) showing that endogenous USP22 and HK2 were not directly bound. (B) Protein and mRNA levels of β‐catenin assessed by western blotting and qRT‐PCR in osteosarcoma cells transfected with shUSP22 or shNC. (C) Protein and mRNA levels of β‐catenin assessed by western blotting and qRT‐PCR in osteosarcoma cells transfected with p‐USP22 or control vector. (D, E) The total and nuclear protein levels of β‐catenin were assessed by western blotting in USP22‐silencing 143B cells (D) or USP22‐overexpression U2OS cells (E). GAPDH and Histone 3 were used as a loading control, respectively. (F) The protein levels of β‐catenin and HK2 were assessed by western blotting in USP22‐overexpression U2OS cells following treatment with shβ‐catenin. (G) Quantification for CCK‐8 assays of USP22‐overexpression U2OS cells transfected with shβ‐catenin. *p < 0.05. (H, I) Quantification (H) and representative images (I) for EDU assays of USP22‐overexpression U2OS cells transfected with shβ‐catenin. *p < 0.05. (J) ECAR of USP22‐overexpression U2OS cells transfected with shβ‐catenin. (K) OCR values of USP22‐overexpression U2OS cells transfected with shβ‐catenin.

As a member of the DUB family, USP22 exerts its function by binding to its substrate. Therefore, to investigate the molecular mechanism by which USP22 activates HK2 in osteosarcoma cells, we verified whether endogenous USP22 and HK2 directly bind to 143B cells. The Co‐IP assay indicated that USP22 and HK2 did not interact directly (Figure 6A). Previous studies indicate that HK2 is a classic downstream gene of β‐catenin in several tumours, including osteosarcoma [41]. Therefore, we hypothesised that USP22 mediates the expression of HK2 by regulating β‐catenin. To confirm this hypothesis, we examined the effects of USP22 knockdown and overexpression on the expression of HK2 and β‐catenin in 143B cells. USP22 knockdown decreased β‐catenin and HK2 protein expression in 143B cells (Figure 6B), whereas USP22 overexpression in U2OS cells increased HK2 and β‐catenin protein expression (Figure 6C). However, changes in USP22 expression did not significantly affect β‐catenin mRNA expression (Figure 6B,C). Free β‐catenin translocate into the nucleus and exerts its transcriptional activity, regulating gene expression. Therefore, we speculated that USP22 may stabilise β‐catenin in osteosarcoma cells and promote its entry into the nucleus. Expectedly, USP22 knockdown decreased β‐catenin levels in 143B cells, whereas USP22 overexpression in U2OS cells increased β‐catenin levels (Figure 6D,E). To further validate our hypothesis, shβ‐catenin‐mediated knockdown of β‐catenin was performed in U2OS cells overexpressing USP22. USP22 overexpression increased HK2 protein levels, cell proliferation, glycolysis rate and OCR (Figure 6F–K). Notably, β‐catenin knockdown did not reverse USP22 overexpression‐induced effects in U2OS cells. Overall, these results indicate that USP22 regulates HK2‐mediated glycolysis in osteosarcoma cells via β‐catenin.

### 3.7 USP22 Stabilises β‐Catenin by Regulating Its Deubiquitination in Osteosarcoma Cells

**FIGURE 7:**
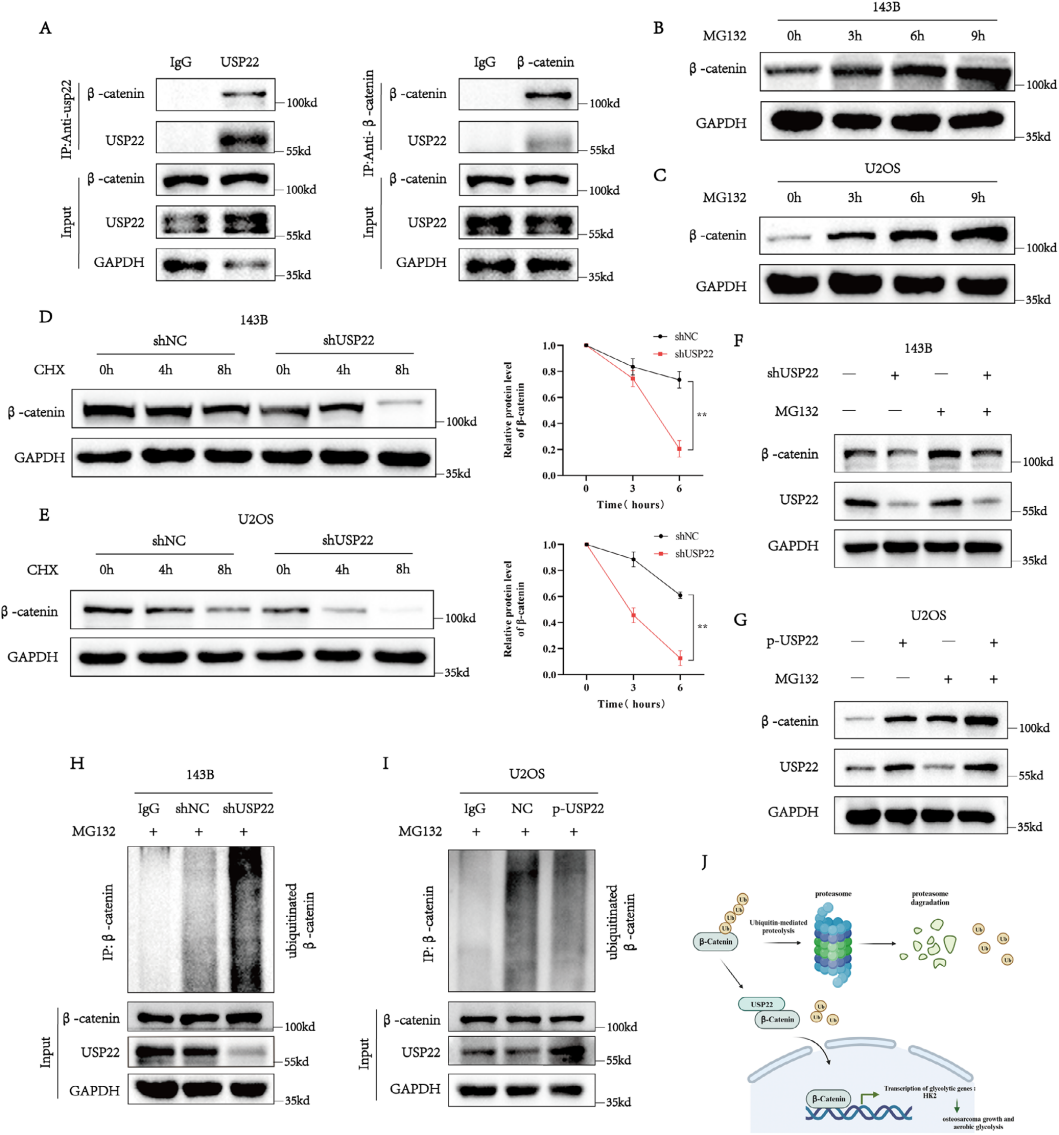
USP22 stabilises β‐catenin by regulating the ubiquitination of β‐catenin in osteosarcoma cells. (A) Co‐immunoprecipitation (Co‐IP) showing direct binding of endogenous USP22 and β‐catenin in osteosarcoma cells. (B, C) Osteosarcoma cells were treated with MG132 (15 μmol/L) for the indicated times, and levels of β‐catenin were determined. (D, E) Representative results of β‐catenin protein level in USP22‐silencing cells. The cells were treated with cycloheximide (CHX, 100 mg/mL) for indicated time points were subjected to western blot analysis. (F, G) OS cells transduced with shUSP22 (F) or p‐USP22 (G) were treated with 10 μM MG132. Cells were collected at 6 h and immunoblotted with the antibodies indicated. (H, I) Lysates from OS cells transduced with shUSP22 (H) or p‐USP22 (I) were immunoprecipitated with the anti‐Ub and immunoblotted with the anti‐β‐catenin. Cells were treated with MG132 for 6 h before collection. (J) Proposed model by which ubiquitin‐specific protease USP22 promotes osteosarcoma growth and aerobic glycolysis by upregulating HK2 via stabilisation of β‐catenin.

USP22 regulates the expression of HK2 by regulating β‐catenin; therefore, we investigated the molecular pathways by which USP22 regulates β‐catenin. As shown in Figures 6B and 6C, USP22 expression did not affect the β‐catenin mRNA level, indicating that USP22 regulates β‐catenin post‐transcriptionally. Given that USP22 is a deubiquitination enzyme, it may be involved in β‐catenin degradation via the ubiquitin proteasome pathway [40]. Therefore, we hypothesised that USP22 regulates β‐catenin expression via deubiquitination. To verify our hypothesis, we examined whether endogenous USP22 can directly interact with β‐catenin in osteosarcoma cells and found that there was a considerable interaction between USP22 and β‐catenin (Figure 7A). Additionally, there was a significant increase in endogenous β‐catenin accumulation in osteosarcoma cells following treatment with the proteasome inhibitor MG132 (Figure 7B,C). Additionally, USP22‐knockdown osteosarcoma cells were treated with cycloheximide. USP22 knockdown increased the degradation of β‐catenin (Figure 7D,E). Additionally, treatment with the proteasome inhibitor MG132 restored β‐catenin protein levels in osteosarcoma cells regardless of the USP22 expression level (Figure 7F,G). Moreover, USP22 knockdown significantly increased β‐catenin ubiquitination in 143B cells (Figure 7H). In contrast, USP22 overexpression in U2OS cells decreased the ubiquitination level of β‐catenin (Figure 7I). Overall, these results indicate that β‐catenin in osteosarcoma cells is regulated by USP22 via the ubiquitin proteasome pathway.

## 4 Discussion

Research advances in cancer research have improved the treatment of patients with osteosarcoma; however, patient prognosis remains relatively poor. Aerobic glycolysis, which increases metabolic reprogramming, has been identified as a tumour marker and is crucial for the development and progression of malignancies [42]. Previous studies have shown significant changes in aerobic glycolysis in osteosarcoma cells [43]. Therefore, a comprehensive understanding of the pathophysiology of osteosarcoma from a metabolic perspective may lead to the development of novel treatment approaches.

In the present study, we examined the expression and role of USP22 in osteosarcoma in vivo and in vitro. USP22 is among 11 genes that are strongly correlated with tumour invasion, chemotherapy resistance and prognosis [44]. USP22 stimulates the growth and incidence of tumours and is significantly expressed in several malignancies, including breast, colorectal and liver cancers [27]. USP22 is involved in various biological characteristics of tumours, and its molecular function is consistent with its carcinogenic effect. However, the precise function and molecular mechanism of USP22 in osteosarcoma remain unexplored. Metabolic transformation is an adaptive means for tumour cells to survive and develop in relatively malnourished microenvironments. For instance, Circ‐CTNNB1 promotes m6A modification by interacting with RBM15 in osteosarcoma cells to drive glucose metabolism reprogramming [7]. In this study, USP22 was highly expressed in osteosarcoma tissue samples and cell lines. In vitro and in vivo studies have demonstrated that USP22 stimulates aerobic glycolysis in osteosarcoma cells, thereby promoting osteosarcoma progression. This discovery strengthens our understanding of the role and mechanism of USP22 in the reprogramming of tumour cell metabolism. Therefore, an in‐depth analysis of the mechanism underlying increased aerobic glycolysis in osteosarcoma cells may provide new treatment opportunities for osteosarcoma.

As the rate‐limiting enzyme in glycolysis, HK2 plays a crucial role in initiating the glycolytic pathway, and is highly expressed in several types of tumours [34, 35, 36]. Furthermore, HK2's major function in controlling energy metabolism serves as the foundation for its control of tumour growth [37, 45]. For instance, Cao et al. discovered that the Warburg effect and the incidence of breast cancer were both promoted by HK2‐mediated circular RNA circRNF20 [34]. N6 methyladenosine METTL3 has been shown by Wang et al. to upregulate HK2 protein expression through altering YTHDF1/HK2, increasing levels of glycolysis and encouraging the onset and progression of cervical cancer [36]. Furthermore, DeWaal et al. [35] showed that in hepatocellular carcinoma, HK2 depletion causes oxidative phosphorylation and inhibits glycolysis. The aforementioned research suggests that HK2, a crucial molecular bridge for the transition of energy metabolism and the fast proliferation of different tumour cells, is a prime candidate for osteosarcoma research. Furthermore, HK2 is among the important glycolysis‐related enzymes whose expression can be controlled by USP22, according to a recent study [31], however the precise molecular mechanism remains unknown. It is obvious that more research is needed because these results alone are inadequate. In addition, USP22 stabilises HIF‐1α through deubiquitination, and its expression promotes hypoxia and stem cell induced glycolysis in liver cancer cells [26]. Therefore, we speculated that the effect of USP22 on glycolysis in osteosarcoma cells might be related to HK2. qRT‐PCR and western blot analysis showed that USP22 and HK2 were highly expressed in osteosarcoma tissues and were positively correlated. Additionally, USP22 knockdown decreased the proliferation and glycolytic rate of the osteosarcoma cell line 143B. However, the effects of USP22 knockdown were partially counteracted by HK2 overexpression, suggesting that USP22 promotes glycolysis and cell proliferation in osteosarcoma by upregulating the expression of HK2. Further analyses were performed to identify the precise molecular pathway through which USP22 regulates HK2 expression. β‐catenin plays a crucial role in tumour and non‐tumour disorders, especially in tumour proliferation and metastasis, metabolism and immunity [39]. Notably, aberrant β‐catenin activation may contribute to tumour occurrence and progression and an increased cancer mortality rate [46]. HK2 is a classic downstream gene of β‐catenin, regulating tumour glucose metabolism [41]; however, its role in osteosarcoma is unclear. In the present study, USP22 knockdown markedly reduced the protein levels of HK2 and β‐catenin and strongly suppressed the proliferation and glycolytic rate of osteosarcoma cells. In contrast, USP22 overexpression significantly increased the protein levels of HK2 and β‐catenin and promoted the proliferation and glycolytic rate of osteosarcoma cells. Overall, this study uncovered a novel mechanism through which USP22 regulates HK2 expression by mediating β‐catenin protein levels.

Furthermore, we investigated the molecular mechanisms by which USP22 regulates β‐catenin expression. Research findings indicate that β‐catenin is involved in various cancers and is stabilised by DUB. For example, USP8 promotes proliferation, invasion and tumour cell stemness in liver cancer by stabilising β‐catenin mediated ferroptosis [40]. USP8 is currently the first deubiquitinase in the USP family that can stabilise β‐catenin. It is still unknown whether β‐catenin can be stabilised by other deubiquitinases, which has sparked our interest in further exploration. Additionally, USP22 only plays a role in promoting the nuclear entry of β‐catenin in pancreatic cancer [47]. Therefore, we speculated that USP22 may be regulated via the ubiquitination modification of β‐catenin. Expectedly, we demonstrated an interaction between USP22 and β‐catenin. Additionally, USP22 knockdown shortened the half‐life of β‐catenin, and MG132 treatment blocked the effects of USP22 knockdown or overexpression on β‐catenin. Moreover, USP22 knockdown increased β‐catenin ubiquitination in MG132‐treated cells, which was significantly decreased by USP22 overexpression. Based on these results, it could be concluded that USP22 regulates β‐catenin in osteosarcoma cells.

## 5 Conclusion

USP22 promotes the progression of osteosarcoma by enhancing glycolysis and cell proliferation, both in vivo and in vitro. Additionally, HK2 mediates USP22‐induced regulation of glycolysis in osteosarcoma cells. Mechanistically, USP22 regulates HK2 by deubiquitinating and stabilising β‐catenin expression, thereby promoting glycolysis in osteosarcoma cells (Figure 7J). Therefore, targeting the USP22/β‐catenin/HK2 axis may be an effective strategy for osteosarcoma treatment.

## Author Contributions

**Shenliang Chen:** software (equal), validation (equal), visualization (equal), writing – original draft (equal). **Xin Hu:** resources (equal), validation (equal). **Xuan Yi:** supervision (equal), writing – review and editing (equal). **Xueqiang Deng:** conceptualization (equal), writing – review and editing (equal). **Ting Xiong:** data curation (equal). **Yanghuan Ou:** supervision (equal). **Shuaigang Liu:** validation (equal). **Chen Li:** conceptualization (equal). **Xiaohua Yan:** conceptualization (equal). **Liang Hao:** conceptualization (equal), project administration (equal), resources (equal), writing – review and editing (equal).

## Ethics Statement

In the study, the use of human tissue was clearly approved by the Ethics Committee of the Second Affiliated Hospital of Nanchang University. The use of All animal experiments were approved by the Animal Experimental Ethics Committee of the Second Affiliated Hospital of Nanchang University.

## Consent

Each participant signed written informed consents prior to the study.

## Conflicts of Interest

The authors declare no conflicts of interest.

## Supporting information


**Figure S1.** (A, B) The mRNA(A) and protein (B) levels of USP22 in U2OS cells after transfection with p‐USP22 or Vector.
**Figure S2.** (A) Quantification of EdU assays of 143B cells stably transfected with shUSP22 in the presence or absence of p‐HK2.

## Data Availability

The datasets generated and analysed during the current study are available from the corresponding author upon reasonable request.
