# Supplementary figures and images for: USP22 Promotes Osteosarcoma Progression by Stabilising β‐Catenin and Upregulating HK2 and Glycolysis
Source: J Cell Mol Med. 2024 Dec 11;28(23):e70239. doi: 10.1111/jcmm.70239 (PMC11633763; doi:10.1111/jcmm.70239)

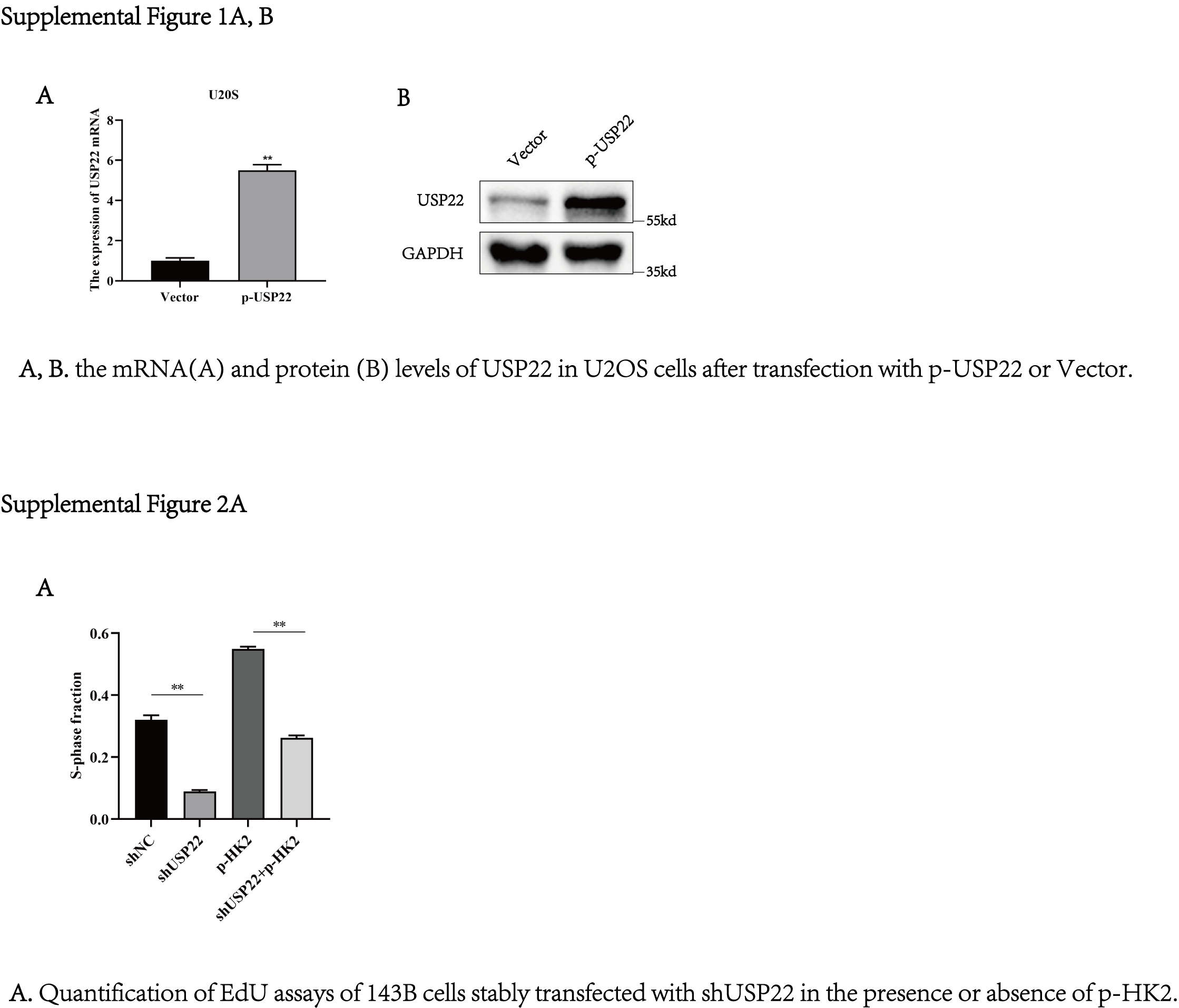

Supplement: Supplementary file 1 — Figure S1. (A, B) The mRNA(A) and protein (B) levels of USP22 in U2OS cells after transfection with p‐USP22 or Vector. Figure S2. (A) Quantification of EdU assays of 143B cells stably transfected with shUSP22 in the presence or absence of p‐HK2. [file JCMM-28-e70239-s001.tif]
